# Parathyroid hormone enhances gap healing and osseointegration in orthopedic porous coated titanium implants: a correlative micro-computed tomographic, histomorphometric and biomechanical analysis

**DOI:** 10.1186/s12891-021-04917-y

**Published:** 2022-01-03

**Authors:** Xinlin Gao, Yang Meng, Dingjun Hao, Hao Liu

**Affiliations:** 1 Department of Spine Surgery, Honghui Hospital, Xi’an Jiaotong University Health Science Center, Xi’an, Shaanxi China; 2 Department of Orthopedics, West China Hospital, Sichuan University, Chengdu, Sichuan China

**Keywords:** Parathyroid hormone, Osseointegration, Cervical disc arthroplasty, Implant

## Abstract

**Background:**

Parathyroid hormone, with its anabolic effect on bone formation, has shown excellent outcomes of curing postmenopausal osteoporosis as well as enhancing osseointegration around orthopaedic and stomatologic implants.The purpose of the present study is to test if low-dose intermittent PTH (1–34) treatment could achieve a satisfactory osseointegration in 2-mm peri-implant gaps, as to provide a new idea for improving the stability of such prosthesis, which will be of great clinical value.

**Methods:**

A custom-made titanium implant was implanted on the calvarium of New Zealand White rabbits. 48 male rabbits were randomly divided into control and PTH group. PTH group received subcutaneous injection of PTH (20 μg/day, 5 days/week). Animals were sacrificed at 4 and 8 weeks after surgery. Quantitative micro-computed tomography, histology and biomechanical pull-out testing were performed to evaluate the gap healing at implantation site.

**Results:**

Analysis of micro-computed tomography demonstrated that PTH group achieved more new bone formation in 2-mm gaps and on bone-implant interface. Quantitatively, significant differences were observed between two groups in regard to BIC and BV/TV at each time-point. Histological staining revealed that PTH group had a superiority in trabecular number, thickness, separation and better osseointegration compared to control group. As for biomechanical pull-out testing, PTH group also showed significant improvement of ultimate force than control group.

**Conclusions:**

Low-dose intermittent administration of PTH for 4 and 8 weeks enhances early osseointegration and fixation of orthopedic implants surrounded by a 2-mm gap in terms of increased bone regeneration and mechanical stability. These findings suggest PTH a potential for reducing the postoperative complications of implants by improving bone healing at peri-implant gaps.

## Background

Parathyroid hormone (PTH) plays an essential role in maintaining calcium homeostasis through its regulation of bone remolding [1]_._ It has a dose-dependent anabolic or catabolic effects on bone formation, that is, continuous high-dose administration results in bone resorption, while low-dose intermittent administration appear to increase bone formation [2–4]. Teriparatide, the first 34 amino acids of PTH, is approved as the only osteogenic drug for treatment of osteoporosis [5]. It has shown to increase bone mineral density and decrease the risk of fracture in postmenopausal osteoporotic women [6, 7]. Also due to its excellent anabolic effect on bone, researchers began to apply it in enhancing osseointegration around orthopedic and stomatologic implants. So far a lot of preclinical trials have been published investigating the efficacy of PTH supplementation on osseointegration of implants [8].

Orthopedic implants are designed in a series of particular sizes based on preclinical bone morphometry. During an operation, size selection of the implant is determined by the test of model together with the surgeons’ experience. Different from custom-sized 3D-printed (three dimensional-printed) implants, this pattern has advantages of convenience and cost-effectiveness. However, due to individual bone variation, the mismatch of size between implant and individual unavoidably generate peri-implant gaps. Last secure fixation requires additional new bone formation, rapidly bridging the peri-implant gaps and new bone within the porosity of the implant surface, besides interfacial bone-implant attachment^9^. Many studies have investigated the effect of PTH on various type of implant fixation, however, only one study published aimed to investigate the effects of PTH on peri-implant gap healing in normal cancellous bone. Daugaard et al [9] inserted a custom-made titanium implant in the proximal tibia of canine to create 1-mm peri-implant gaps as simulation to that after total joint replacement surgery, results showed that PTH successfully overcome the obstacle of 1-mm gap and achieve a positive effect on fixation. To our knowledge, no studies are available about the effects of PTH on peri-implant gaps over 1 mm.

**Fig. 1:**
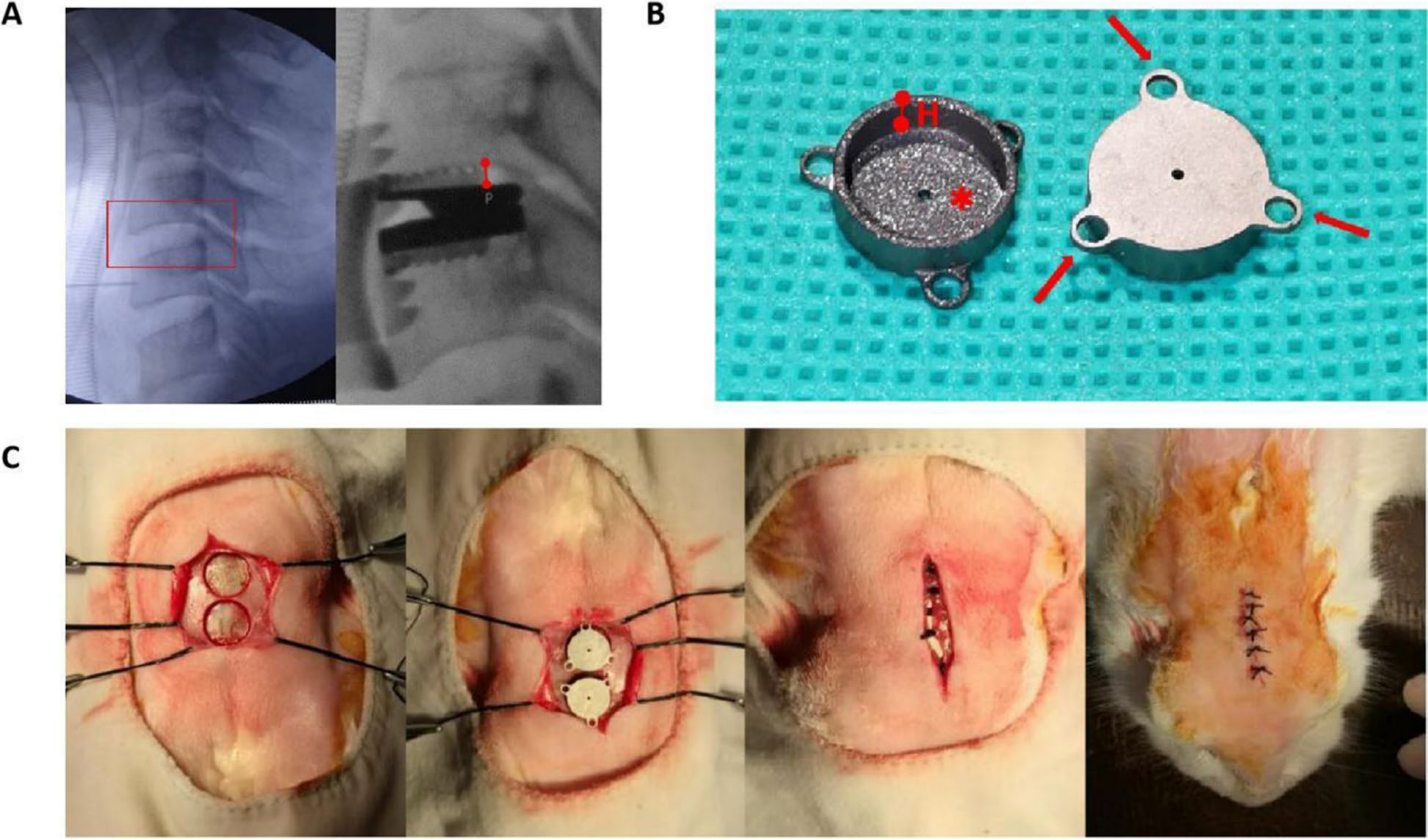
**A** Left: Intraoperative lateral X-ray image of cervical spine before insertion of prosthesis (Red box: Operational segment with an obvious doom-shape inferior endplate). Right: Intraoperative lateral X-ray image of operational segment after insertion (Red line segment: Max distance between endplate and footprint). **B** The custom-made titanium implant used in the present study (Arrow: Rings designed as the fixture of implant in biomechanical testing; H: The depth of the inner cavity is designed as 2 mm; *:Porous hydroxyapatite-coated inner surface. **C** Surgical procedures showing a sequential of exposure and drilling, insertion and closure in layers

Gap size more than 1 mm is sufficient to delay or prevent substantial healing without intervention during a 4-week observation period according to a former study [10]. In order to obtain the immediate stability and avoid long-term nonunion, implants are designed using exact- or press-fit insertion to eliminate the existence of gaps in implantation. Among all the orthopedic operations, cervical disc arthroplasty (CDA) may face the most challenging environment in the aspect of gap healing because of the severe mismatch between the dome-shape inferior endplate of vertebral body and the plat footprint of disc prostheses (Fig. 1A). Many quantitative morphometric studies assessing the depth of inferior endplate in cervical spine are now available in literature [11–13]. Given the clinical prevalence in CDA of gaps between footprint and inferior endplate, the performance of PTH on gap healing is worth expecting.

The purpose of the present study is to test if low-dose intermittent PTH (1–34) treatment could achieve a satisfactory osseointegration in 2-mm peri-implant gaps, as to provide a new idea for improving the stability of orthopedic implants surrounded by gaps, which will be of great clinical value.

## Materials and methods

### Animals and experimental design

The Institutional Animal Care and Use Committee at the West China Center of Medical Sciences, Sichuan University, Chengdu, Sichuan province granted approval for the investigation. Conduct of experimentation involved in the research followed the recommendations of the Animal Research: Reporting In Vivo Experiments (ARRIVE) guidelines [14], all procedures were preformed in accordance with relevant guidelines. 48 four-month-old male New Zealand White rabbits, each weighing approximately 3 kg, were included in this study. After implantation, rabbits were randomly divided into two groups: Control and PTH. PTH group received subcutaneous injection of PTH (20 μg/day, 5 days/week) (Forteo®, Eli Lilly, Indianapolis, Indiana, USA) at posterior neck until sacrifice. While the control group received same dosage of vehicle (sterile saline). Animals were weighed every 7 days and injection volumes were adjusted accordingly. The animals were kept in a purpose-designed room for experimental animals and were fed with a standard laboratory diet. At the timepoint of 4 weeks after implantation, 12 rabbits in each group were sacrificed and their calvarium with implants were harvested. Half specimens in each group (*N* = 6) were stored in 10% neutral buffered formalin waiting for the evaluations of micro-CT scan and the subsequent histological observation, the other half (N = 6) were stored at − 20 °C for the biomechanical test. Same procedures were repeated at 8 weeks after implantation.

### Implants

External diameter and length of the custom-made, cylindrical titanium implants (Ti_6_Al_4_V ELI, ASTM-F136; ZHSM, Shenyang, China) are 12 mm and 3 mm respectively (Fig. 1B). The diameter of the inner cavity was 10 mm and the depth was 2 mm (mean [SD] 2.0[0.08] mm) which was designed according to the results of former studies about morphometry of inferior endplate in cervical spine [11–13, 25–29]. The surface of the inner cavity was plasma sprayed porous hydroxyapatite (HA) coating (thickness 59um, crystallinity 58%). Three rings extending from the side wall of cylindrical implants were designed to enable its attachment to the biomechanical test device.

### Surgery

Surgical procedures were performed under aseptic conditions and under general anesthesia with sodium pentobarbital (40 mg/kg, i.v.). A medial incision was made on the skin and the subsequent galea aponeurotica over the calvarium. After exposure a defect was prepared using a round, 10 mm in diameter, low-speed drill with cooling of saline irrigation drip. Stopping drilling when a 2-mm depth is ensured to preserve the underlying bone tissue as much as possible. After drilling, the implant was inserted and incisions was closed in layers. Suture of the galea aponeurotica could produce strain on implant to keep it on position. All the animal surgeries were performed by one single experimenter(Dr.Gao). Prophylactic antibiotics (penicillin, 400,000 IU, i.m. qd) was administered for the first three postoperative days (Fig. 1C).

### Micro-CT

**Fig. 2:**
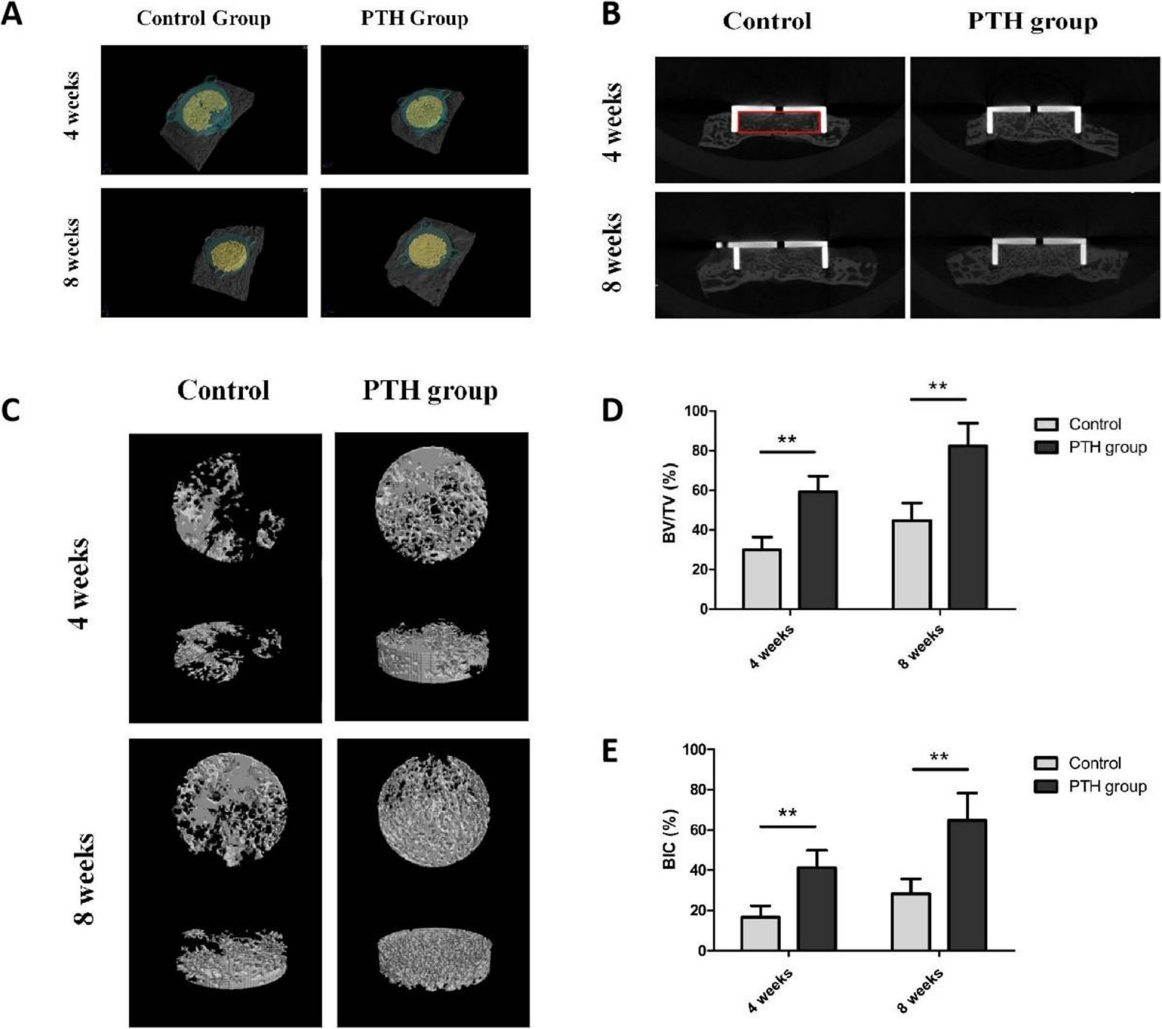
Analysis of micro computed tomography. **A** Represntative CT images of three-dimensional reconstruction in each group (Green: implant; Yellow: new bone formation inside the implant; Gray: calvarium). **B** Images of coronal section showing new bone formation inside the implant (Rex box: region of interest, ROI). **C** Three-dimensional reconstruction of the new bone formation in ROI. **D-E** Quantitative analysis of bone-implant contact (BIC) and bone volume density (BV/TV) in ROI. (Error bars represent SD, ^**^P<0.01, control group versus PTH group, *n* = 6)

The specimens were scanned on a micro-CT system (Perkin Elmer, Massachusetts, USA) in the following scanning parameters: Xray kV: 90 kV, μA: 88 μA, scan time: 14 min, field of view: 25 mm, pixel size: 5.0 μm. The data analysis and volume rendering procedures were perfermed by CTAn and Ctvox softwares (Bruker, Wisconsin, USA). The region of interests (ROI) included the inner cylindrical cavity of the implant (Fig. 2A). The calculated morphometric parameters within ROI included bone volume density (BV/TV) and bone-implant contact (BIC). The top internal surface was traced manually and expressed as a total endplate area pixel count, the regions of trabecular contact were subsequently traced and quantified in pixels and then expressed as a percentage of the total endplate area (BIC = pixels in bone contact area/ gross total endplate area). This evaluation parameter was widely used to describe the osseointegration of prosthesis in previous researches [15, 16]. The above quantitative analysis was performed by one independent observer blinded to the divided groups.

### Histological observation

Specimens were fixed in 10% paraformaldehyde phosphate buffer saline. After micro-CT scan, the specimens were decalcified in nitric acid and then implants were gently removed. The remaining specimens were then dehydrated in a graded series of ethanol (75, 85, 95 and 100%) and finally embedded in paraffin. Serial coronal sections were cut in a thickness of 4–5 μm from the center of the cylindrical-shaped newly-generated bone tissue and mounted on microscope slices, stained in a systematic fashion of Hematoxylin and Eosin (HE). The image acquisition is performed using NanoZoomer® digital pathology system including a virtual slide scanner NanoZoomer S60 and the matched software NDP.view2 (Hamamatsu Photonics, Hamamatsu, Japan).

### Biomechanical testing

Specimens were unfrozen in PBS 48 h prior to the biomechanical pull-out testing (AG-IS, Shimadzu, Japan). The specimen was placed on a metal base and fixed by two mechanical magnetic elements PD-102 (Oukai, Dongguan, China). Three rings, designed to be the fixture of the implant, were firmly connected to the testing system by metal hook and wire (Fig. 4A). A displacement velocity of 2 mm/minute was used. The pull-out testing was performed until the implant was completely separated from the bone, the load-displacement curve was automatically drawn and the ultimate force was determined. Testing was done blinded by one experienced technician.

### Statistical analysis

Statistical analyses were carried out using SPSS statistical software version 19.0 (SPSS, Chicago, Illinois, USA) and analyzed for statistical significance by one-way ANOVA. All data values were presented as mean ± standard deviation (SD) and significance was defined as P<0.05. Pearson correlation coefficient was used to evaluate the relationship between micro-CT measurements and biomechanical parameter.

## Results

### Micro-CT

The ROI of the Micro-CT analysis was the inner cavity of the implant as shown in Fig. 2A. The median coronal section planes (Fig. 2B) and three-dimensional reconstruction images (Fig. 2C) were used describe the new bone formation in ROI. These images demonstrated that both PTH administration and longer experimental period enhanced bone regeneration. At each follow-up timepoint, PTH group showed more new bone formation in the gaps and bone-implant interface, namely achieved a better osseointegration compared to the control group. Quantitatively, significant differences were observed between two groups in regard to BIC and BV/TV at 4 and 8 weeks after surgery (P<0.01, Fig. 2D-E).

### Histology

**Fig. 3:**
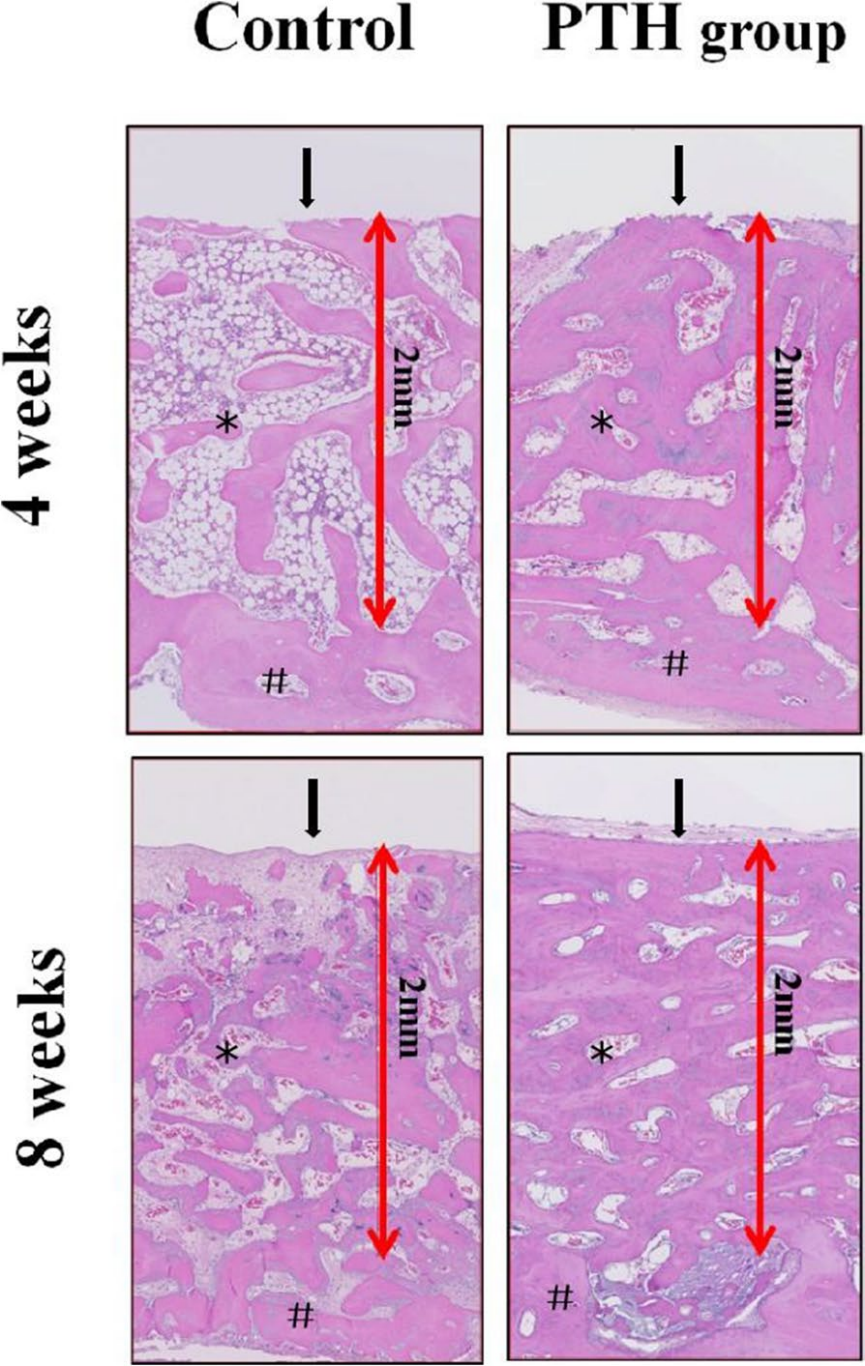
Histology. Photomicrographs of representative histological samples (hematoxylin/eosin staining, magnification 10×) (*: new bone formation; #: former bone tissue; arrow: Bone-implant interface)

A qualitative histological examination was shown in Fig. 3. In general, images in PTH group showed higher bone volume and trabecular number as well as lower trabecular separation than control group. In the aspect of BIC, more bone tissue appeared at the bone-implant interface in PTH group. As for the comparison between two observation periods, images at 8 weeks showed a superiority in trabecular number, thickness, separation, bone volume and osseointegration than that at 4 weeks, indicating a better gap healing occurred with the extension of time.

### Biomechanical testing

**Fig. 4:**
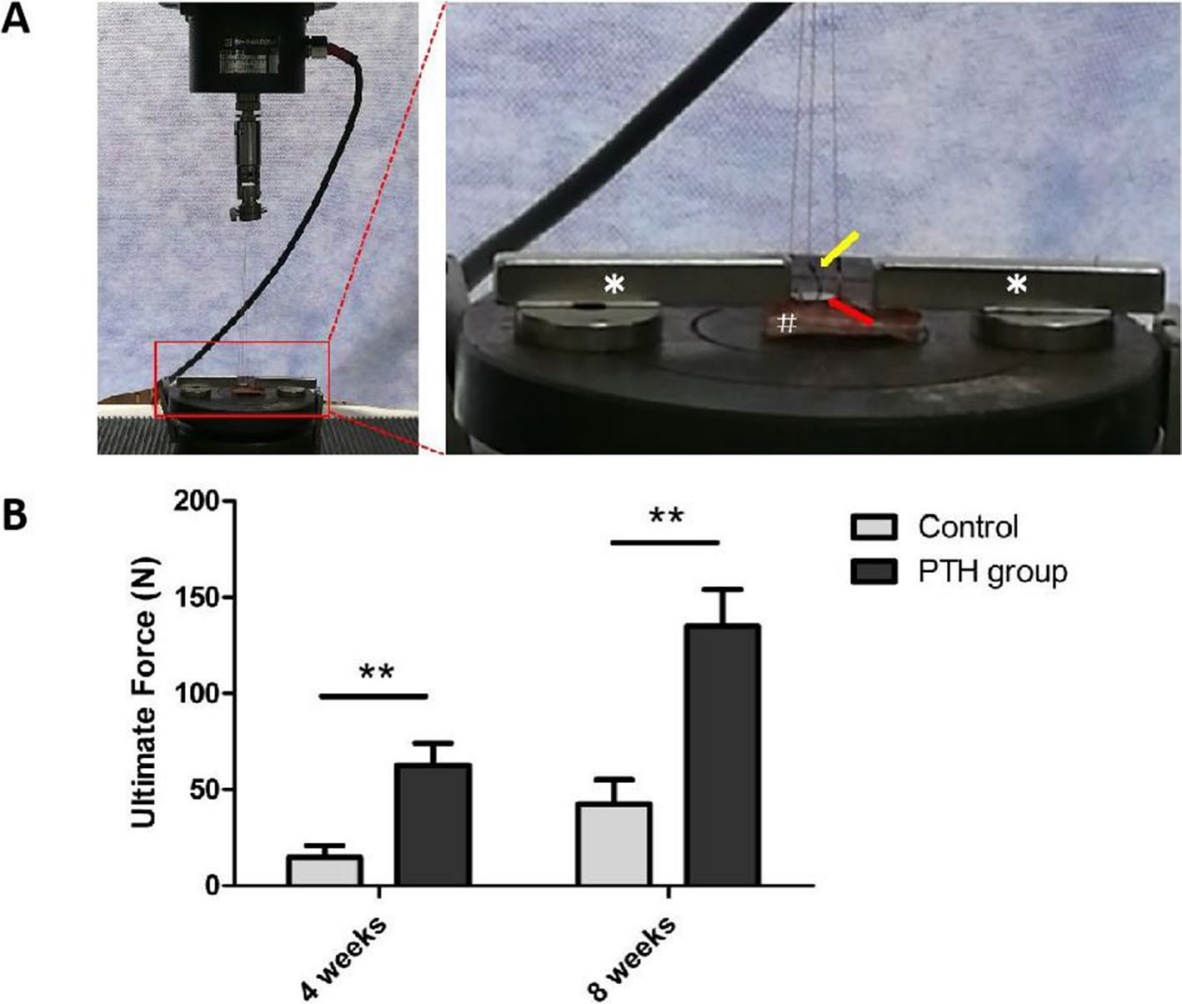
Analysis of biomechanical pull-out testing. **A** Image (*: mechanical magnetic bar using to fix specimen; #: calvarium fragment; yellow arrow: Metal hook and wire using to connect implant and testing device; red arrow: implant). **B** Results (Error bars represent SD, ^**^P<0.01, control group versus PTH group, n = 6)

Results of the biomechanical pull-out testing were shown in Fig. 4. At the timepoint of both 4 and 8 weeks after surgery, significant differrences of the ultimate force were observed between two groups (P<0.01). This parameter in PTH group was 4.3-fold, 3.2-fold higher than that in control group at 4 weeks and 8 weeks, respectively. According to the analysis of Pearson correlation coefficient, both BIC and BV/TV showed significant correlation coefficients with the ultimate force (*r* ^2^ = 0.780, 0.689, respectively, P<0.01).

## Discussion

Cervical disc arthroplasty is an alternative surgical procedure to anterior cervical discectomy and fusion (ACDF) in selected patients suffering cervical degenerative disc disease with a superiority of preserving the segmental motion and thus avoiding the adjacent segment degeneration. After more than 10 years′ clinical application, long-term follow-up results of CDA are now becoming available in literature. Most researches reported favorable clinical outcomes and confirmed the success of its core idea — protecting the adjacent segment. However, complications related to instability including subsidence, migration and dislocation are still commonly seen clinically, with an reported occurrence rate of 3–10%, 8.2–10.9% and 0.5–3.06% [17–20], respectively. Such complications inhibit the recovery of symptoms after surgery and may lead to an ending of re-operation. Loss of stability is a consequence of multiple factors, including low bone density, improper endplate preparation and inadequate load distribution [17]. In addition, insufficient osseointegration plays a key role in long-term instability [21]. In fact, CDA prosthesis may face the most challenging environment than other orthopedic implants in the aspect of gap healing, that is because the mismatch between the dome-shape inferior cervical endplate and the flat footprint of prostheses. As described by Beer et al [22], the contact proportion of endplate and footprint was merely 10.49% in average after CDA surgery, this would not only decrease the contact area between bone and fixation component, but also make the gap healing more difficult - both increase the risk of postoperative instability.

With the development of radiological technology and related image processing software, studies concentrating on the quantitative morphometry of the subaxial cervical vertebrae endplate are now available in literature. Former anatomic morphometry was conducted by radiographing the living body or autopsy specimen and then measuring the parameters [23, 24]. The parameter describing the inferior endplate of the cervical spine was merely the width and depth. Now benefit from the computed tomography (CT) and finite element method, a more accurate morphometry can be used to describe the concavity of inferior endplate. Such studies, replacing the former sagittal and coronal section with three-dimensional reconstruction, emphasized the existence of concavity and described it using more parameters such as the concavity depth, the location of concavity apex, the curvature radius, the endplate area and slope [11–13], [25–29].

Among all kinds of the operations located in cervical spine, the existence of the concavity at inferior endplate probably have the greatest influence on CDA surgery. Because the footprint has a direct contact with the endplate, and stability of the prosthesis highly depend on its fixation with the endplate. Martin et al [30] assessed the accuracy of match between cervical vertebrae and several common cervical disc prostheses, the results confirmed a common existence of size mismatch. This mismatch may lead to subsidence, loosening, heterotopic ossification and biomechanical failure which decrease the efficacy and safety of the surgery. So far multiple efforts had been taken to improve the osseointegration of cervical disc prosthesis including using titanium alloy, porous surface, multiple kinds of coating, improved fixture component. But complications related to instability seemed still common according to the follow-up outcomes. Considering the remarkable capacity of PTH for promoting osseointegration in the published studies, we thought PTH administration may be an alternative to overcome the difficulty of gap healing in CDA surgery.

As for the animal model, the cervical spine of goats was the most suitable choice because of its similarity with human’s in anatomic morphology and biomechanical characteristic [31, 32]. But complicated operation and high expenditure limited its usage in large sample size. The mandible of canines was widely used in stomatology to observe the osseointegration of implants [33–35], while the physiological environment in oral cavity was quite different from that of cervical spine. The rabbit calvarial defect model was a novel model to evaluate the bone regeneration in defects [36, 37]. It has advantages of simple operation, convenient feeding as well as low expenditure, thus were commonly used in large-sample experiments. Based on above, we finally choose the rabbit calvarial defect model in this study.

The present study indicated that PTH could effectively promote the bone regeneration at 2-mm peri-implant gaps. Similar conclusions were also observed in previous studies concentrating on the effect of PTH on osseointegration of orthopedic implants. Daugaard et al [9] inserted a cylindrical porous coated titanium alloy implant in a concentric 1-mm gap in cancellous bone of tibia in canines to examined the effect of PTH on implant fixation. After 4 weeks both bone volume and mechanical implant fixation were improved. The author concluded that PTH has a positive effect on implant fixation in regions where gaps exist in the surrounding bone. Gabet et al [38] inserted a implant in proximal tibial metaphysis of postorchiectomy (ORX) rats to assess whether PTH enhanced titanium implant osseointegration in low-density bone. The results showed that maximal values of ROI, BV/TV, Tb.Th in PTH-treated ORX rats exceeded significantly those measured in the implantation site of untreated ORX controls. Biomechanical values including ultimate force, stiffness and toughness showed the same tendency. Those findings demonstrated that PTH effectively stimulate implant anchorage in low-density trabecular bone, and thus have the feasibility to improve the prognosis of implants in low-density trabecular sites, it may show potency in conducting bone healing of large peri-implant gaps in clinical use.

## Conclusion

Low-dose intermittent administration of PTH for 4 and 8 weeks enhances early osseointegration and fixation of orthopedic implants surrounded by 2-mm gaps in terms of increased bone regeneration and mechanical stability. These findings suggest PTH a potential for reducing the postoperative complications of orthopedic implants by improving bone healing at peri-implant gaps. In the future, studies concentrating on dose and adverse reaction of PTH, larger or critical size of gaps, different kinds of implants, as well as clinical experiments were expected.

## Data Availability

The datasets used and/or analysed during the current study available from the corresponding author on reasonable request.

## Abbreviations

CDA: cervical disc arthroplasty; ACDF = anterior cervical discectomy and fusion; PTH parathyroid hormone; PBS = phosphate buffer saline; HA = hydroxyapatite; ROI region of interests; BV/TV = bone volume / total volume; BIC = bone-implant contact
HE: hematoxylin and eosin; SD = standard deviation; CT = computed tomography; HO heterotopic ossification.

## References

[1] Silva BC, Bilezikian JP (2015). Parathyroid hormone: anabolic and catabolic actions on the skeleton. Curr Opin Pharmacol.

[2] Ejersted C, Andreassen TT, Nilsson MH (1994). Human parathyroid hormone (1–34) increases bone formation and strength of cortical bone in aged rats. Eur J Endocrinol.

[3] Dempster DW, Cosman F, Kurland ES (2001). Effects of daily treatment with parathyroid hormone on bone microarchitecture and turnover in patients with osteoporosis: a paired biopsy study. J Bone Miner Res.

[4] Hock JM, Gera I (1992). Effects of continuous and intermittent administration and inhibition of resorption on the anabolic response of bone to parathyroid hormone. J bone Min Res.

[5] Brixen KT, Christensen PM, Ejersted C (2004). Teriparatide (biosynthetic human parathyroid hormone 1-34): a new paradigm in the treatment of osteoporosis. Basic Clin Pharmacol Toxicol.

[6] Orwoll E, Scheele WH, Paul S (2001). Brief therapy with recombinant human parathyroid hormone (1–34) increases lumbar spine bone mineral density in men with idiopathic or hypogonadal osteopenia. J Bone Miner Res.

[7] Neer RM, Arnaud CD, Zanchetta JR (2001). Effect of parathyroid hormone (1–34) on fractures and bone mineral density in postmenopausal women with osteoporosis. N Engl J Med.

[8] Javed F, Al Amri MD, Kellesarian SV (2016). Efficacy of parathyroid hormone supplemention on the osseointergration of implants: a systematic review. Clin Oral Inveat.

[9] Daugaard H, Elmengaard B, Andressen T (2011). Parathyroid hormone treatment increases fixation of orthopedic implants with gap healing: a biomechanical and Histomorphometric canine study of porous coated titanium alloy implants in Cancellous bone. Calcif Tissue Int.

[10] Soballe K (1993). Hydroxyapatite ceramic coating for bone implant fifixation. Mechanical and histological studies in dogs. Acta Orthop Scand Suppl.

[11] van der Houwen EB, Baron P, Veldhuizen AG (2010). Geometry of the intervertebral volume and vertebral endplates of the human spine. Ann Biomed Eng.

[12] Chen H, Zhong J, Tan J (2013). Sagittal geometry of the middle and lower cervical endplates. Eur Spine J.

[13] Feng H, Fang XY, Huang DG (2017). Quantitative morphometric study of the subaxial cervical vertebrae endplate. Spine J.

[14] Percie du Sert N, Ahluwalia A, Alam S (2020). Reporting animal research: explanation and elaboration for the ARRIVE guidelines 2.0. PLoS Biol.

[15] McAfee PC, Cunningham BW, Orbegoso CM (2003). Analysis of porous ingrowth in intervertebral disc prostheses: a nonhuman primate model. Spine..

[16] Hu N, Cunningham BW, McAfee PC (2006). Porous coated motion cervical disc replacement: a biomechanical, histomorphometric, and biologic wear analysis in a caprine model. Spine..

[17] Shi R, Li J, Liu H (2014). Clinical comparison of 2 implantation systems for single-level cervical disk replacement. Orthopedics..

[18] Li J, Liang L, Ye XF (2013). Cervical arthroplasty with discover prosthesis: clinical outcomes and analysis of factors that may influence postoperative range of motion. Eur Spine J.

[19] Anderson PA, Sasso RC, Riew KD (2008). Comparison of adverse events between the Bryan artificial cervical disc and anterior cervical arthrodesis. Spine..

[20] Ozbek Z, Ozkara E, Arslantas A (2017). Implant migration in cervical disk Arthroplasty. World Neurosurg.

[21] Lebl DR, Cammisa FP, Girardi FP (2012). The mechanical performance of cervical total disc replacements in vivo: prospective retrieval analysis of prodisc-C devices. Spine..

[22] de Beer N, Scheffer C (2012). Reducing subsidence risk by using rapid manufactured patient-specific intervertebral disc implants. The spine journal : official journal of the North American Spine Society.

[23] Gilad I, Nissan M (1985). Sagittal evaluation of elemental geometrical dimensions of human vertebrae. J Anat.

[24] Manohar MP, Joanne D, Vijay G (1991). Cervical human vertebrae quantitative three-dimensional anatomy of the middle and lower regions. Spine..

[25] Zhao SC, Hao DJ, Jiang YH (2016). Morphological studies of cartilage endplates in subaxial cervical region. Eur Spine J.

[26] Yao Q, Yin P, Khan K (2018). Differences of the morphology of subaxial cervical spine endplates between Chinese and white men and women. Biomed Res Int.

[27] Panjabi MM, Duranceau J, Goel V (1991). Cervical human vertebrae: quantitative three-dimensional anatomy of the middle and lower regions. Spine J.

[28] Tan SH, Teo EC, Chua HC (2004). Quantitative three dimensional anatomy of cervical, thoracic and lumbar vertebrae of Chinese Singaporeans. Eur Spine J.

[29] Kim MK, Kwak DS, Park CK (2007). Quantitative anatomy of the endplate of the middle and lower cervical vertebrae in koreans. Spine J.

[30] Martin T, Sebastian H, Michaela G (2013). Footprint mismatch in total cervical disc arthroplasty. Eur Spine J.

[31] Cain CC, Fraser RD (1995). Bony and vascular anatomy of the normal cervical spine in the sheep. Spine..

[32] Wilke HJ, Kettler A, Wenger KH (1997). Anatomy of the sheep spine and its comparison to the human spine. Anat Rec.

[33] Al-Sulaimani AF, Mokeem SA, Anil S (2013). Peri-implant defect augmentation with autogenous bone: a study in beagle dogs. J Oral Implantol.

[34] Kold S, Rahbek O, Toft M (2005). Bone compaction enhances implant fixation in a canine gap model. J Orthop Res.

[35] Rossi F, Botticelli D, Pantani F, et al. Bone healing pattern in surgically created circumferential defects around submerged implants: an experimental study in dog. Clin Oral Implants Res. 2012;23:–41. 10.1111/j.1600-0501.2011.02170.x 21443594

[36] Kim S, Hwang Y, Kashif M (2016). Evaluation of bone regeneration on Polyhydroxyethyl-polymethyl methacrylate membrane in a rabbit Calvarial defect model. In Vivo.

[37] Shao H, Ke X, Liu A (2017). Bone regeneration in 3D printing bioactive ceramic scaffolds with improved tissue/material interface pore architecture in thin-wall bone defect. Biofabrication..

[38] Gabet Y, Muller R, Levy J (2006). Parathyroid hormone 1-34 enhances titanium implant anchorage in low-density trabecular bone: a correlative micro-computed tomographic and biomechanical analysis. Bone..

